# Emulating Agricultural Disease Management: Comparing Risk Preferences Between Industry Professionals and Online Participants Using Experimental Gaming Simulations and Paired Lottery Choice Surveys

**DOI:** 10.3389/fvets.2020.556668

**Published:** 2021-01-18

**Authors:** Eric M. Clark, Scott C. Merrill, Luke Trinity, Gabriela Bucini, Nicholas Cheney, Ollin Langle-Chimal, Trisha Shrum, Christopher Koliba, Asim Zia, Julia M. Smith

**Affiliations:** ^1^Social Ecological Gaming and Simulation Lab, University of Vermont, Burlington, VT, United States; ^2^Department of Plant and Soil Science, University of Vermont, Burlington, VT, United States; ^3^Gund Institute for Environment, University of Vermont, Burlington, VT, United States; ^4^Complex Systems Center, University of Vermont, Burlington, VT, United States; ^5^Department of Computer Science, University of Vermont, Burlington, VT, United States; ^6^Department of Community Development and Applied Economics, University of Vermont, Burlington, VT, United States; ^7^Department of Animal and Veterinary Sciences, University of Vermont, Burlington, VT, United States

**Keywords:** experimental games, veterinary diseases, decision making, behavior, experimental economics, health economics, data science

## Abstract

Mitigating the spread of disease is crucial for the well-being of agricultural production systems. Implementing biosecurity disease prevention measures can be expensive, so producers must balance the costs of biosecurity investments with the expected benefits of reducing the risk of infections. To investigate the risk associated with this decision making process, we developed an online experimental game that simulates biosecurity investment allocation of a pork production facility during an outbreak. Participants are presented with several scenarios that vary the visibility of the disease status and biosecurity protection implemented at neighboring facilities. Certain rounds allowed participants to spend resources to reduce uncertainty and reveal neighboring biosecurity and/or disease status. We then test how this uncertainty affects the decisions to spend simulation dollars to increase biosecurity and reduce risk. We recruited 50 attendees from the 2018 World Pork Expo to participate in our simulation. We compared their performance to an opportunity sample of 50 online participants from the survey crowdsourcing tool, Amazon Mechanical Turk (MTurk). With respect to biosecurity investment, we did not find a significant difference between the risk behaviors of industry professionals and those of MTurk participants for each set of experimental scenarios. Notably, we found that our sample of industry professionals opted to pay to reveal disease and biosecurity information more often than MTurk participants. However, the biosecurity investment decisions were not significantly different during rounds in which additional information could be purchased. To further validate these findings, we compared the risk associated with each group's responses using a well-established risk assessment survey implementing paired lottery choices. Interestingly, we did not find a correlation in risk quantified with simulated biosecurity investment in comparison to the paired lottery choice survey. This may be evidence that general economic risk preferences may not always translate into simulated behavioral risk, perhaps due to the contextual immersion provided by experimental gaming simulations. Online recruitment tools can provide cost effective research quality data that can be rapidly assembled in comparison to industry professionals, who may be more challenging to sample at scale. Using a convenience sample of industry professionals for validation can also provide additional insights into the decision making process. These findings lend support to using online experimental simulations for interpreting risk associated with a complex decision mechanism.

## 1. Introduction

Disease outbreaks across livestock production systems can have devastating economic consequences. Porcine Epidemic Diarrhea Virus (PEDv), for example, is a coronavirus that costs the U.S. industry an estimated $900 million to $1.8 billion per year (1–3). Here, biosecurity refers to the initiative to stem the spread of disease in agriculture (4), which include a set of tools for disease prevention (i.e., vaccines) along with sanitary regulations and protocols that can mitigate disease transmission across production systems. Increased biosecurity reduces disease transmission between producers (5). However, biosecurity tools and practices vary in cost and perceived efficacy (6, 7). Hence, supply chain managers must balance the costs of biosecurity investments with the expected benefits of reducing the risk of infection. Our aim is to investigate the strategies used to achieve this balance, by quantifying risk mitigation behaviors associated with economic investment in biosecurity. Our research approach focuses on applying digital simulations for studying this decision making process.

Experimental gaming simulations, a branch of “serious gaming,” are tailored interfaces that leverage software from game design to recreate a complex decision mechanism (8–12). Here we use simulations to collect decision making data and analyze responses with respect to various visual stimuli that are designed to communicate risk. This is our lab's primary tool for studying human behavior and how risk preference may influence the spread of disease among agricultural supply chains (9, 13–15).

Although biosecurity has been shown to reduce disease prevalence, widespread adoption of biosecurity varies, possibly due to uncertainty in efficacy and return on investment (7). Our experimental gaming simulation tests risk preference with regards to several scenarios in which disease prevalence and neighboring biosecurity visibility are varied. By injecting different types of uncertainty into experimental game simulations, we can explicitly observe response to uncertainty and how it may change as risk communication strategies adapt.

Our previous study (14) analyzed the risk associated with biosecurity investment decisions across a multitude of disease outbreak scenarios. Using a sample of 1,000 participants from an online survey recruitment marketplace, Amazon Mechanical Turk (MTurk), we found three prominent risk strategies—risk tolerant, opportunistic, and risk averse—by analyzing responses with regard to disease threat. We then investigated how information uncertainty affects the decision making process, by varying the visibility of the disease spread and biosecurity protection across each simulated population of farms (13, 14). Among this sample, we found that high visibility in disease spread led to more risk averse behaviors while high visibility in biosecurity status led to more risk tolerance. We also investigated how risk preference may differ among a sample of industry professionals and stakeholders. We attended the 2018 World Pork Expo, the world's largest pork industry trade show attended by thousands of producers and industry professionals (https://worldpork.org/about-expo/all-about-expo). Here, we recruited 50 attendees to complete our experimental gaming simulation. Their performance was then compared to 50 MTurk recruits, in addition to the 1,000 recruits sampled in (14). When aggregating across all experimental outbreak scenarios, we did not find a significant difference in biosecurity investment risk distributions. In this work, we aim to further investigate potential differences in risk preference among World Pork Expo participants and online recruits from MTurk. We compare biosecurity investment decisions during each set of experimental scenarios as well as the willingness to spend economic resources to reduce uncertainty.

We also compare our sampled participants' behavior using a well-established risk assessment survey using paired lottery choices (16). This context-free, multiple price list approach (17) measures risk aversion with respect to economic preference by varying the probability of a high and low payout. Using their choices, participants can then be categorically grouped into “Risk Seeking,” “Risk Neutral,” or “Risk Averse.” The main difference between this paired lottery choice assessment and our experimental gaming simulations is the context surrounding the decision making process. The paired lottery choice assessment attempts to measure underlying preferences in a purely economic trade-off. Whereas the economic risk management associated with biosecurity investment decisions are specifically framed in the context of agricultural outbreak mitigation. We compare risk preferences associated with lottery choices against simulated biosecurity investment strategies between World Pork Expo participants and MTurk recruits.

Several studies investigated how risk aversion delineated using this paired lottery choice assessment (16) have compared to real world behaviors. Experimental market trading behaviors were found to correlate with paired choice lottery risk aversion (18). Negative health related behaviors including cigarette smoking, heavy drinking, obesity, and seat-belt non-compliance were found to be anti-correlated with surveyed risk aversion (19). Similarly, a generalized self-assessment risk survey could predict surveyed lottery risk aversion (20). These contexts based risk aversion measures were actually in some cases better predictors of malbehaviors in comparison to multiple price list assessments (21). This difference in performance may be attributed to additional background information captured regarding the individuals' preferences using context based measures of risk aversion.

Providing contextual background to the studied decision making process can have a pronounced effect on risk mitigation. For example, the domain specific risk-taking scale (DOSPERT) (22) is a context driven risk assessment questionnaire which has shown promising results in characterizing risk averse behaviors across several content domains. This flexible measurement is useful for categorizing risk with respect to content areas in which individuals may exhibit various levels of risk aversion depending on the framing. Context based risk assessments can provide additional insights into behavioral response and how risk may fluctuate with respect to situational framing.

Our experimental gaming simulation exemplifies this initiative for capturing contextually driven risk mitigation behaviors. We found that risk aversion characterized by multiple choice lottery assessment differed from risk associated with behaviors in our simulated environment. This supports the argument that context driven risk assessment may be more appropriate for identifying behavioral risk regarding specific domains. Capturing these nuanced behaviors may prove illusive in the lens of traditional multiple price list risk assessment frameworks.

Our previous findings (14) did not detect a difference in biosecurity investment decisions between World Pork Expo attendees and recruits from MTurk. However, aside from biosecurity investment, other behavioral aspects may differ between these cohorts. Our current study investigates how Pork Expo attendees and online recruits from Amazon Mechanical Turk may diverge in their decision making with respect to each tested experimental scenario. Although we previously found the overall distributions of risk were comparable between Pork Expo and MTurk groups, it is also important to highlight where there may be differences in simulated behavior. This validation process is necessary when recruiting large convenience samples from online survey marketplaces.

Along with the ability to invest in biosecurity, our simulation allowed participants to purchase information to reduce uncertainty in the decision making process by revealing infection and biosecurity status of neighboring facilities. Due to expo attendees' industry knowledge and expertise, we may expect to find a measurable difference in their willingness to purchase information in comparison to a opportunity sample of online recruits. We also investigate whether Pork Expo participants' biosecurity investment strategies and experimental earnings (i.e., their performance) differed across each particular experimental scenario. This leads to our first tested hypothesis:

(H1): Participants with industry knowledge will invest more experimental resources to procure information and reduce uncertainty in the decision making process.

In addition, we compare risk associated with simulated biosecurity investment to risk aversion measured using a paired lottery choice survey (16). The paired lottery choice survey has a well-defined payoff function where economic benefit and risk are clearly established during each decision. Our experimental simulation's risk decision tradeoff is more obscured by visual assessment, assumptions regarding disease spread, and protection offered by neighboring facilities' biosecurity implementation. Our study investigates how these two risk assessment frameworks align. (23) found real-world farmer production risk, as formulated by (24), correlated with paired lottery choice risk aversion. We may expect that participants who invest more simulated resources in biosecurity would behave with more risk aversion in the paired lottery choices. We formulate this hypothesis (which we later reject) as:

(H2): More investment in simulated biosecurity will correspond with more risk aversion in the paired lottery choice assessment.

## 2. Methods

We created a digital application to assess the impact of economic consequences on decisional risk. The experimental gaming simulation and risk assessment survey were engineered using the Unity Development Platform. The final application was deployed using WebGL (25) and hosted on the University of Vermont's web server, where simulation decision data were stored in a relational database. The 2018 World Pork Expo participants completed the experiment in our booth using provided tablet computers. The 50 online participants were contracted using Amazon Mechanical Turk and compensated through Amazon with a base pay of $2.00 USD for successfully completing the assignment along with a bonus payment based upon their simulation performance. On average MTurk participants earned an additional $7.93 (σ = *$*4.98) and completed the experiment in 10.92 min (σ = 4.80 min), after the introductory on-boarding. We paid the participants at the World Pork Expo a higher rate to bolster attendance and interest. Pork Expo participants earned on average $16.11 (σ = *$*4.26) over 15.97 min (σ = 4.91 min) to complete the simulation and survey. These monetary incentives are crucial to our experimental design and have been found to increase salience and immersion in the decision making process (26, 27).

### 2.1. Biosecurity Investment Experimental Game

Our methods were derived from the online experimental simulation featured in (13). The experimental game allowed participants to allocate simulated resources toward biosecurity investment during disease outbreak scenarios with disease spreading across a production system. Each experiment began with an introductory slide show, which framed the study design and informed the player of the game mechanics and interface. The game introduction slideshow can be found in (14). We obtained informed consent from each participant, during our prepared introductory slide presentation prior to the experiment. These practices were accepted by the University of Vermont Institutional Review Board concerning experiments using human participants (University of Vermont IRB # CHRBSS-16-232-IRB).

**Figure 1:**
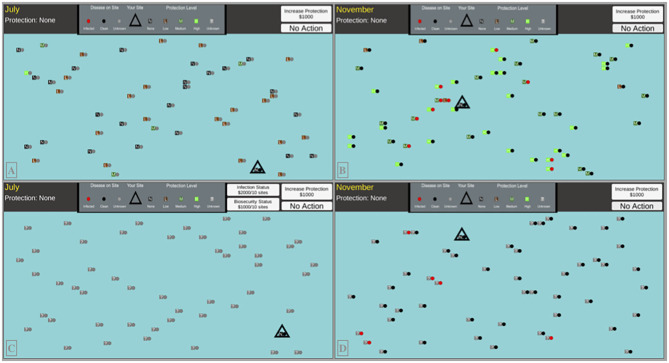
Simulation interfaces for a sample of our experimental treatment scenarios: **(A)** High Disease Uncertainty, No Biosecurity Uncertainty **(B)** No Uncertainty **(C)** Full Uncertainty, Option to pay to reveal disease and biosecurity status **(D)** High Biosecurity Uncertainty, No Disease Uncertainty. Every combination of these information treatments were tested.

Each of our 50 industry participants and MTurk online participants completed 32 simulated outbreak scenarios (i.e., 6 month decision years), for up to 192 decisions per person (depending upon infections). We collected a total of 18,716 decisions to compare (9437 Pork Expo; 9279 MTurk). Numbers differ slightly because decisions during a round would be truncated if the participant's facility became infected. Each round of decision making featured adaptations to the interface and/or information regarding the infection status and biosecurity allocation among the population of farms (see Figure 1). The participant is in charge of a single production facility, surrounded by 50 computer-controlled facilities. Every round consists of six decision months in which players have the choice to invest their simulation dollars in more biosecurity for their own facility. The simulated dollars earned were converted to U.S. currency after completion of the experiment. Online recruits were compensated at a rate of $1 USD to $23,500 simulation dollars, on top of their base pay of $2.00 USD for completing the assignment. Participants from the World Pork Expo were paid a rate $1 USD to $12,000 simulation dollars.

Biosecurity investment reduces the probability of infection. Players could sequentially increase their biosecurity status once per each of the six decision months at the cost of $1,000 simulation dollars, from “None” to “Low” to “Medium” and a maximum of “High.” In our simulation, each successive level of biosecurity implemented reduces the probability of infection by 25%. If the player did not wish to invest in biosecurity, they could choose “No Action” to continue to the next decision month. At the end of each decision month, the infection could progress to any production facility with a varying infection rate probability (*p*_*inf*_ = 0.15) that decreased with distance from the infection source. Explicitly, the raw probability of transmission between an infected facility and a clean facility separated by distance, *D*, would be calculated as $\frac{p_{inf}}{D^{2}}$, which was then adjusted by the clean facility's biosecurity level. If the player's facility became infected, the round would immediately end and the player would lose $25,000 simulation dollars. For each consecutive round, the participant's biosecurity status was reset to “None” and the infection and neighboring set of farms were re-initialized by randomly reassigning the geographical positions and biosecurity status of each facility.

One quarter of all rounds presented full visibility of the infection status and neighboring biosecurity to the player. The other 75% of tested treatments injected uncertainty into this decision mechanism by cloaking the infection status and system-wide biosecurity configuration. Additionally, certain rounds featured the capability to purchase more information regarding the infection status and/or biosecurity allotment for 10 neighboring facilities. The cost for revealing this information varied between either $1,000 or $2,000 simulation dollars. Examples of our user interface (UI) for a sample of our information treatment types are given in Figure 1.

### 2.2. Paired Lottery Choice Risk Assessment

**Figure 2:**
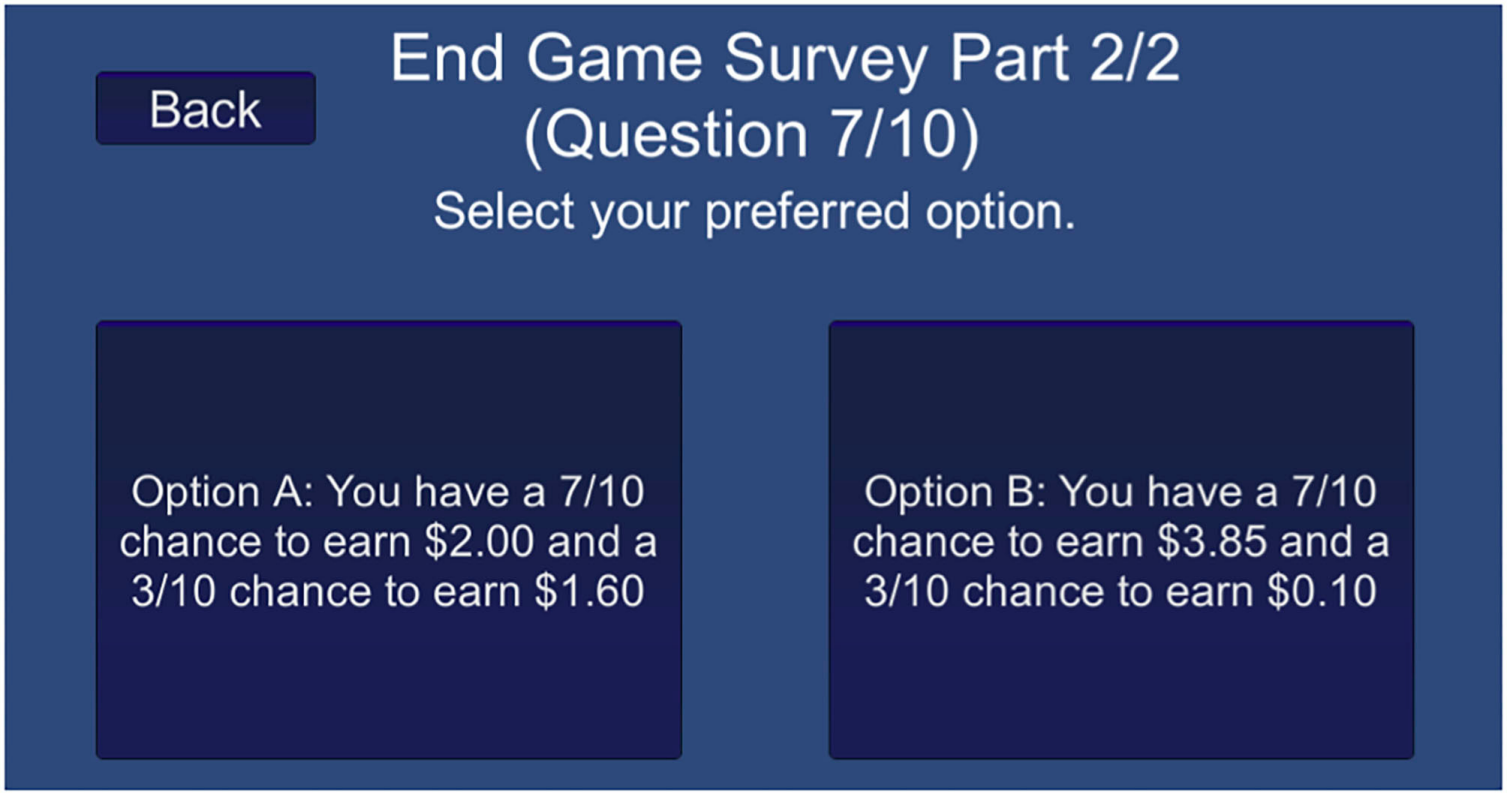
Paired lottery choice risk Assessment interface: a sample of the menu design of the paired lottery choice survey.

We created a digital version of the risk assessment paired lottery choice survey featured in (16). Participants are instructed to choose their preference across ten distinct paired lottery choices. Each pair of choices features a safer “Option A” in comparison to a more risky “Option B,” which has a higher pay gap between the two reward probabilities. For example, the first choice features Option A as a 1/10 chance to earn $2.00 and a 9/10 chance to earn $1.60, while Option B presents a 1/10 chance to earn $3.85 and a 9/10 chance to earn $0.10. Here, only the most risk tolerant of individuals may consider choosing Option B over Option A. This probability gap between the higher and lower payment for each choice sequentially increased, such that the more risky option becomes more viable as the survey progresses (i.e., the expected payoff of Option B becomes greater than the expected payoff of Option A). By choice 7, the payout probability is now 7/10 for each high reward and 3/10 for the low reward (see Figure 2). Here, it becomes somewhat ambiguous which is the more appropriate option, creating an interesting risk dilemma to study.

A natural “crossover point” occurs where a rational individual may move from choosing the safer Option A to a more risky Option B. We quantify risk using the ratio that “Option A” was chosen over the ten paired lottery choices. Participants had the option to revise their choices up until their final decision, after which a random number generator was used to select one of their decisions and then determine their reward. Randomly implementing one of their choices insured that every choice was incentive-compatible, meaning that participants had an incentive to reveal their true preferences. Our digital interface of this paired lottery survey featured in our simulation is provided in the Supplementary Material.

In this portion of the study, we also compensated volunteers from the 2018 World Pork Expo a slightly higher rate: Option A $2.00 USD or $1.60 USD; $3.85 USD or $0.10 USD. Online recruits were paid either $0.60 or $0.50 for Option A or were paid $1.10 or $0.05 for Option B.

### 2.3. Statistical Methods

In our simulation, each decision whether or not to invest resources in biosecurity has an associated financial risk. We implemented a biosecurity investment rating, *R*_*i*_ defined in (14) for each participant *i*. This is the weighted average of the player facility biosecurity status across a set of decision months. For each round, the biosecurity status (0 = “None,” 1 = “Low,” 2 = “Medium,” 3 = “High”) of the player's facility is tallied and then normalized by the total number of decisions. For example, suppose for one round, participant *j* invested experimental dollars to obtain “Low” biosecurity in month 3 of 6 and then “Medium” biosecurity on month 5 of 6. Then *R*_*j*_ = 1.0 = $\frac{1}{6}\cdot \left[0+0+1+1+2+2\right]$. More biosecurity reduces the risk of infection. Hence, a higher biosecurity investment rating is associated with more risk averse behaviors, which is an indication of participants' risk preference.

Risk aversion in the paired lottery choice survey was measured using the number of “safe” (Option A) choices registered by each participant and then normalized by their total number of decisions. For example, if a participant chose Option A 4 times out of 10, their surveyed risk aversion score is 0.4, which would be considered “risk neutral” behavior. More than 4 safe choices correspond to more risk averse behavior while less than 4 choices designate risk tolerant behavior. We chose this risk metric to be consistent with (16).

Statistics were performed using Python 2.7 SciPy statistical libraries (28). The two-sample Kolmogorov–Smirnov (KS) test (29) was implemented to compare risk lottery preferences for consistency with (16). To quantify differences in risk aversion with respect to biosecurity investment ratings between sampled participants, we performed one-tailed Mann–Whitney *U*-tests (30). We chose the *U*-test since in our previous study (14) we found that the biosecurity investment ratings failed the D'Agostino and Pearson's test for normality (31, 32). Preferential risk distributions were displayed using violin plots (33). We tested statistical correlations between risk associated with simulation decisions and the risk preference lottery using Spearman's rank (*r*_*s*_), correlation coefficient (34).

A demographic comparison between the World Pork Expo and MTurk cohorts are given in the Supplementary Materials. Demographic categorical frequencies comparing age, gender and education between samples were differentiated using the Chi Square (χ^2^) statistical test (35). We did not find evidence that demographics effected the decision-making process.

## 3. Results

We compare the decisions from 50 industry professionals and stakeholders from the 2018 World Pork Expo to 50 MTurk online participants. Additionally, we measured participants' risk preferences using the paired lottery choice assessment distributed in our exit survey, and noted how these preferences contrast with risk behaviors quantified using simulated biosecurity investment management.

### 3.1. Biosecurity Investment Simulation

We compared the distributions of biosecurity investment ratings between each set of participants using two-tailed Mann–Whitney *U*-tests across each treatment. We did not detect a difference in the distribution of biosecurity investment decisions between Pork Expo participants and MTurk recruits. We summarize these findings in Table 1. We also compared the session profit (i.e., overall simulation dollars earned) between samples and did not find a significant difference in earnings: PE [μ = 163,740.00; σ = 48,997.07; min = 44,000; max = 258,000], MTurk [ μ = 172,620.00; σ = 38,612.89; min = 91,000.00; max = 263,000.00]: U = 1,095.0, *p* = 0.2867 (two-tailed).

**Table 1:** Experimental treatment comparison.

**Treatments**	**Biosecurity** ****(μ, σ)****		**U**	**p-value**
	**PE**	**MTurk**		
All	(1.38, 0.67)	(1.43, 0.72)	1214.5	0.68
Din Visible	(1.45, 0.67)	(1.56, 0.71)	1157.0	0.42
Bio Visible	(1.41, 0.65)	(1.23, 0.72)	1445.0	0.24
Bio Hidden	(1.33, 0.73)	(1.56, 0.75)	1046.5	0.12
Bio Reveal	(1.39, 0.71)	(1.46, 0.78)	1211.0	0.66
Dis Hidden	(1.37, 0.71)	(1.43, 0.76)	1178.0	0.51
Dis Reveal	(1.35, 0.72)	(1.36, 0.78)	1266.5	0.97

*Biosecurity Investment ratings per experimental scenario delineated for each sample of 50 participants, Pork Expo (PE) and Mechanical Turk (MTurk). Comparisons are given for each set of Disease (Dis) and Biosecurity (Bio) visibility treatments, along with rounds in which participants can spend resources to reveal information regarding the infection and/or biosecurity. Using two-tailed Mann–Whitney U-tests, no significant differences were found in biosecurity investment across each set of experimental scenarios*.

Although we did not find a significant difference between biosecurity investment distributions or performance, we did, however, detect a difference in the willingness to purchase information regarding biosecurity and infection status. To investigate hypothesis (H1) we tested two price levels, {$1,000, $2,000}, for revealing the biosecurity rating or infection status of 10 neighboring facilities. Comparatively, both groups invested more resources in infection status information: Expo total $401,000 (averaging $8020/person) v. MTurk total $316,000 (averaging $6,320/person). Overall, less resources were spent on biosecurity information, however with a larger division between cohorts: Expo total $256,000 (averaging $5,120/person) v. MTurk total $119,000 (averaging $2,380/person). We then quantified this difference in choices per participant between samples using the Mann–Whitney *U*-test.

**Figure 3:**
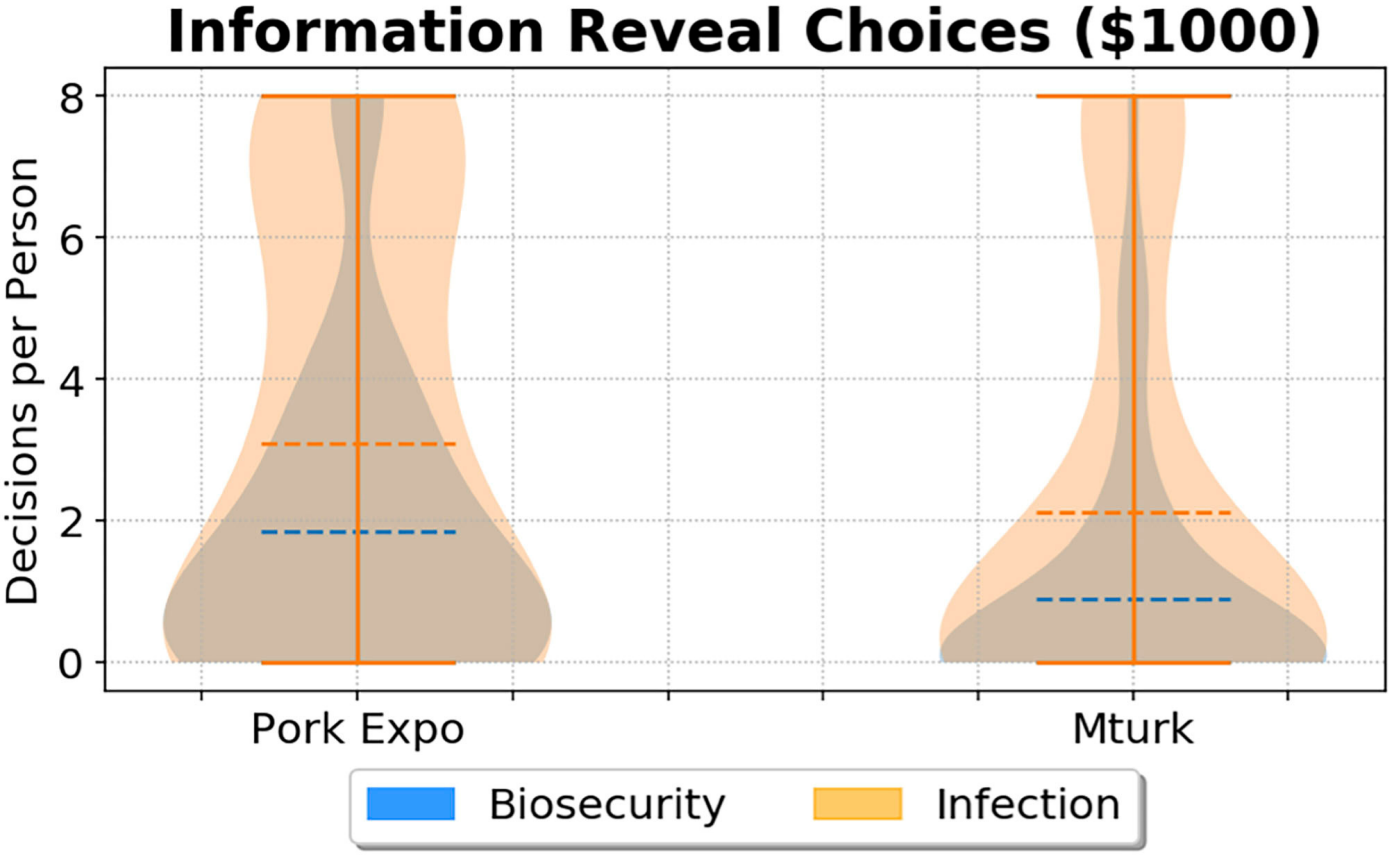
Information reveal choice comparison ($1,000/reveal) between 2018 World Pork Expo (PE) participants and Amazon Mechanical Turk (MTurk). For each group, per person choices to reveal biosecurity (blue) are overlaid with choices to reveal disease statuses (orange). The dotted line denotes the mean of each distribution. For this experimental treatment, Pork Expo participants paid to reveal information more often than MTurk participants.

We found that the Pork Expo group chose to spend significantly more money to reveal both biosecurity and infection data than MTurk participants, when the price was $1,000 per reveal. The difference in biosecurity spending was highly significant (*p* = 0.0013), while the difference in infection reveals was marginally significant (*p* = 0.0487). However, if we consider the power of the disease status result by adjusting the *p* value to control for the false discovery rate (36) due to performing 4 statistical tests, we find, (*p*_*adj*_ = 0.078), suggesting more sampling may be required for verification. However, the difference in biosecurity information spending between groups was highly significant, even after adjustment (*p*_*adj*_ = 0.005). In Figure 3, violin plots show each distribution of information reveal choices ($1,000) per participant for disease status (orange) and biosecurity (blue). There was no significant difference between groups in spending to reveal biosecurity (*p* = 0.0587) or disease (*p* = 0.2835) information when it cost $2,000 per reveal (i.e., twice the price of increasing biosecurity). The results from each Mann–Whitney *U*-test per experimental treatment are given in Table 2. These results support hypothesis (H1) that participants with industry knowledge will invest more resources to reduce information uncertainty, given the stipulation that pricing motivates differences in this decision mechanism.

**Table 2:** Reveal treatment comparison.

**Treatment**	**Reveals** ****(μ, σ)****		**U**	**p-value**
	**PE**	**MTurk**		
Inf $1,000	(3.10, 3.07)	(2.12, 2.93)	1481.5	**0.0487**
Inf $2,000	(2.46, 2.88)	(2.10, 2.81)	1330.0	0.2835
Bio $1,000	(1.84, 2.27)	(0.90, 1.77)	1651.5	**0.0013**
Bio $2,000	(1.64, 2.52)	(0.74, 1.23)	1455.5	0.0587

*Choices (per person) to invest economic resources to reveal biosecurity (bio) and infection (inf) status for 2018 World Pork Expo Participants (PE) and Amazon Mechanical Turk (MTurk). We found Expo participants chose (highly) significantly more biosecurity statuses reveals and (marginally) significantly more infection status reveals when the cost was $1,000/reveal (bold). No significant difference was measured when the cost of revealing this information increased to $2,000 simulation dollars*.

We also considered the relationship between information uncertainty reduction and biosecurity adoption. We may expect that more risk aversion would be associated with more choices to reveal information, however this was not supported by the data. Quite conversely, we actually found a moderately strong negative correlation from the MTurk cohort during both infection uncertainty experimental treatments ($1,000,$2,000) for participants who chose at least 1 infection information reveal: [Spearman *rho* = −0.463, *p* < 0.01, *N* = 30]. For these treatments, recruits from MTurk who revealed more infection information tended to adopt less biosecurity. Interestingly, we did not find evidence for this relationship from Pork Expo attendees who chose at least 1 infection information reveal: [Spearman *rho* = 0.014, *p* = 0.93, *N* = 36]. This highlights another interesting difference between these groups. Perhaps, for this subset of industry professionals, investing in additional information did not deter their initiative to situationally adopt biosecurity, whereas MTurk recruits may have been more motivated for maximizing their earnings when investing in reducing uncertainty. We did not find any significant correlation between risk associated with biosecurity adoption and number of biosecurity information reveals from either cohort.

To further investigate this relationship between information uncertainty reduction and biosecurity adoption, we compared the risk preferences of participants who were willing to invest resources in reducing information uncertainty compared to those who opted out and never revealed infection and/or biosecurity statuses. Applying Mann–Whitney *U*-tests, we did not find significant differences in biosecurity adoption between Pork Expo participants who chose to reveal infection/disease information and those who did not. In the MTurk cohort, we did find that recruits who chose to reveal information adopted less biosecurity for the $1,000 biosecurity reveal treatment [*U* = 167, *p* < 0.05:μ_1_ = 1.041, *N*_1_ = 16 v. μ_2_ = 1.584, *N*_2_ = 34] and the $1,000 infection reveal treatment [*U* = 225, *p* < 0.05:μ_1_ = 1.240, *N*_1_ = 26 v. μ_2_ = 1.595, *N*_2_ = 24]. This supports our previous finding that MTurk recruits may have been more reluctant to diminish their potential earnings, while Pork Expo attendees did not sacrifice biosecurity investment when also purchasing additional information.

### 3.2. Paired Lottery Choice Risk Comparison

**Figure 4:**
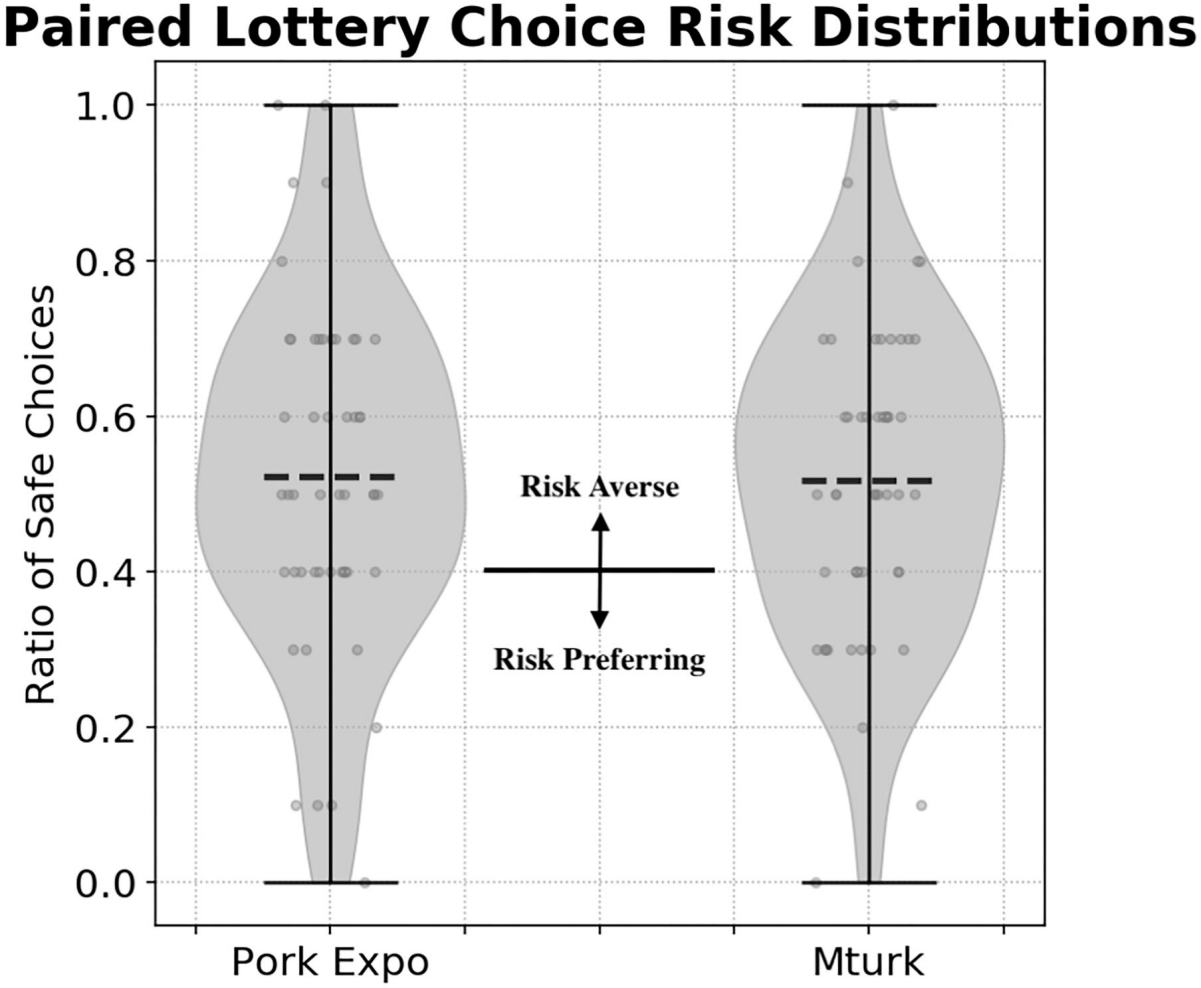
Paired lottery choice survey risk preferences distributions comparing participants from the 2018 World Pork Expo and Amazon Mechanical Turk. The dashed line represents the mean from each distribution. The solid line at 0.4 (i.e., 4/10 safe choices) denotes risk neutral behavior. Making more than 4 safe choices is considered risk averse behavior while less than 4 safe choices is considered risk tolerant.

We compared risk preference distributions between Pork Expo participants [μ = 0.522, σ = 0.219, median = 0.500] and online recruits from Amazon Mechanical Turk [μ = 0.518, σ = 0.199, median = 0.500]. Using a KS test, we did not find a significant difference in risk distributions between each sample : KS [*U* = 0.06, *p* = 0.999, *n*_1_ = *n*_2_ = 50]. In Figure 4, the risk preference distributions compare choices from each sample. As defined in (16), a risk neutral preference is defined as 0.4 (4/10) safe choices (i.e., “Option A”). More than 4 safe choices are deemed as risk averse, while less is considered risk tolerant. Under this metric, the majority of participants in both samples were categorized as risk averse. For Pork Expo participants, 31 (62%) were classified as risk averse, 8 were risk tolerant (16%), and 11 were risk neutral (22%). Mechanical Turk recruits followed a similar distribution: 32 Risk Averse (64%), 11 Risk Tolerant (22%), and 7 were risk neutral (14%).

We also considered the consistency in selections between each group of participants. We would expect that the majority of participants would begin with the safe “Option A” before eventually switching to the more risky “Option B” for the remainder of the survey. In (16) the majority of participants only switch once from “Option A” to “Option B,” however there were cases of multiple switching from their sampled participants. We found the majority of our participants also only switched their responses one time. From the Mechanical Turk group, 2 participants (4%) didn't switch, 44 participants (88%) switched once, 2 participants (4%) switched twice, 1 participant (2%) switched 3 times, and 1 participant (2%) switched more than 3 times. The majority of the Pork Expo attendees also only switched once, however there were more cases of multiples switches: 3 participants (6%) didn't switch, 30 participants (60%) switched once, 6 participants (12%) switched twice, 5 participants (10%) switched 3 times, and 6 participants (12%) switched more than 3 times.

We investigate hypothesis (H2) by comparing risk associated with simulated biosecurity investment to decisions in the paired lottery choice assessment. We did not find a direct correlation between risk preference and biosecurity investment strategies for either sample: (Spearman) Pork Expo *r*_*s*_ = 0.086, *p* = 0.54; MTurk *r*_*s*_ = 0.218, *p* = 0.12; All *r*_*s*_ = 0.142, *p* = 0.156. We considered differences between simulated biosecurity investment with respect to risk classification under the preferential lottery (risk averse vs. risk tolerant in Figure 4), however since the majority in each group were risk averse, we could not reliably test this significantly. Given these results, we couldn't find evidence to support hypothesis (H2), that risk associated with our simulation will correspond with paired lottery choice assessment.

## 4. Discussion and Conclusion

To further explore potential differences in decision making between industry professionals and online recruits, we analyzed their choices regarding paying to gain situational awareness in an experimental game and their decisions in a paired lottery choice survey. We wanted to understand the magnitude of these differences to be able to determine the sample size needed to quantify efficacy of risk communication strategies. We did not find a measurable difference in the distributions of biosecurity investment decisions between World Pork Expo participants and online recruits from Amazon Mechanical Turk across each set of experimental information treatments.

We observed a difference in the willingness of participants to purchase information regarding neighboring infection and biosecurity status. In support of hypothesis (H1), we found participants from the World Pork Expo chose more often to reduce uncertainty in this decision making process. We note cost was a motivator in this decision making process, and at higher costs we found no significant differences in purchasing disease or biosecurity information. The most pronounced difference was the amount of spending by Pork Expo participants on neighboring biosecurity status information. Overall, both groups invested the most resources on reducing uncertainty around disease spread. Pork Expo participants, perhaps due to their industry profiles, had significantly more interest in neighboring biosecurity configurations than MTurk recruits. This distinction may indicate that reducing uncertainty regarding the spread of disease and neighboring biosecurity protection is of particular interest to industry professionals when weighing their risk of infection throughout this decision making process.

Interestingly, we did not find a direct association between lottery risk preference and biosecurity investment decisions, leading us to reject hypothesis (H2) for this sample of participants. One possibility for this lack of consistency in observed behaviors across the two risk assessment methods is the contextual framing that's motivating decisions within the experimental gaming simulation. The lottery risk preference may be contrasted as a measure of pure economic risk preference. Our simulated environment creates a more complex and realistic economic dilemma to tackle. This difference is especially highlighted during rounds that inject additional uncertainty in the decision making process by masking the spread of infection and/or shielding neighboring biosecurity configurations. This provides support for harnessing experimental gaming simulations to study behavioral risk. Experimental gaming simulations may be especially useful for emulating complex decision mechanisms in which nuanced behavioral signals may be difficult to capture using generalized risk assessment survey strategies. Further investigation is needed to accentuate these differences in behavioral responses associated with added contextual framing provided by experimental simulations, in comparison to traditional survey methods for measuring risk preference.

Overall, the distributions of risk associated with our biosecurity investment simulation were statistically comparable to our sample of 50 industry professionals and stakeholders from the 2018 World Pork Expo. Additionally, we found no difference between these two audiences in their performance from the lottery risk preference assessment portion of the experiment. Our findings lend support to using large samples of online recruits, such as MTurk, for identifying general trends in risk attitude and perception. Validation using a sample of participants with related industry knowledge provides confidence for behavioral analyses using experimental gaming simulations.

Potential bias in our results stem from Mechanical Turk participants completing this experiment fully digitally, while participants from the World Pork Expo underwent the simulation in-person during their attendance at the event. The payment scale was the only adjustment between administered digital application interfaces. Differences we are finding in strategies could be affected by Pork Expo attendees' current immersion in the subject material. This may also strengthen our result that the risk distributions with regards to biosecurity investment were similar. We also were limited by our sample size, as recruiting industry professionals is challenging in comparison to online survey marketplaces like MTurk. Hence, it is possible that a larger sampling of participants with an industry background are required to detect differences in behavior. These relationships between industry professionals and online recruits should be further validated when analyzing risk preferences associated with industry-specific decision making.

While comparing decision consistency in the paired lottery choice portion of the risk assessment, we found more Pork Expo participants (≈40%) switched more than once between the safer “Option A” and more risky “Option B” in comparison to the MTurk sample. This is slightly more switching than may be expected. Perhaps this could have been due to survey fatigue, as the Pork Expo attendees were attending the fair recreationally, whereas the MTurk recruits were seeking an employment opportunity. Also, the final lottery choice sets the high payouts for both Option A and Option B at 10/10, so the most rational decision is to choose Option B for this last question. Although the vast majority of participants from both cohorts finished with “Option B,” there was 1 case from the MTurk group and 8 cases from the Pork Expo attendees ending with “Option A.” This difference could also be a sign of potential survey fatigue, or a misunderstanding of the lottery payouts for the final question. Overall the proportion of safe choices between each cohort was comparable and hence this metric for risk aversion was ideal for comparing behavior.

The decision-making data collected from experimental gaming simulations is not only informative in itself but also a valuable resource for disease-spread models lacking a human behavioral component. For example, agent based models (ABMs), (37), are computer simulations that can help forecast outcomes of decisions and interactions of entities (or agents) and their impact on the system. Agent based modeling has been applied to agriculture for producer decision interaction, (38), technology and policy modeling, (39), as well as for water management (40). ABMs can provide insights into epidemiological factors that exacerbate disease spread and their economic impacts on agricultural supply chains (41, 42). Human behavioral components, captured using digital experimental simulations, can then be used to model systemic outbreaks and how disease spread will change as human behavior is altered or risk communication strategies are devised (43). The distributions of behavioral risk observed in our biosecurity experimental gaming simulations can be embedded in these agent based models to test how proportions of risk aversion effect the spread of disease. The model can then be calibrated using real world estimates of viral incidence. Experimental gaming simulations can also provide insights into how individuals may adapt their risk preferences over time. Individuals may learn to become more or less risk averse in response to their simulated outcomes. Studying how different proportions of these risk attitudes effect the spread of disease can help gain insights into forecasting economic impacts and how different risk landscapes impact the well-being of the system. This may be useful to policy regulators interested in developing and testing risk communication strategies that nudge behaviors toward more risk averse disease management practices to help stem the spread of disease.

Experimental gaming simulations are effective tools for examining behaviors surrounding risk associated with agricultural disease mitigation. These readily adaptable simulations allow us to tailor interfaces for capturing subtle behavioral differences while also harnessing population-wide patterns that can be useful for modeling behaviors associated with disease management and prevention. While we do not endorse moving solely to experimental gaming simulations for gathering human behavioral data, our research demonstrates how the additional context provided via simulation can capture distinct behaviors potentially missed using traditional survey methods. Moreover, experimental gaming simulations can increase salience and engagement by immersing participants in real-world dilemmas, thus providing an alternative viewpoint that may more closely approximate real world behavior, and could be used in conjunction with traditional methods to improve our understanding of human decision making processes.

Understanding how behavior in simulated gaming environments translates to real world decisions by industry professionals is an important consideration of this behavioral research. This is still an open question that we'll continue to investigate through our research agenda. Rigorous behavioral validation is challenging due to the vast number of decisions that are tested in our simulation, and by design, farmers are unlikely to have experienced these specific decisions in the real world. The flexibility of the gaming environment to gather behaviors across a multitude of possible scenarios can provide insights into risk management investment strategies that may be difficult to discern using traditional survey instruments. We are also working toward adapting our behavioral games into digital tools and interfaces that may allow industry professionals to emulate their own production system. Creating these decision support tools from our experimental game design may help us better investigate how choices in a simulated environment relate to real-world behavior. This evolution of our experimental gaming simulations into decision support applications may provide insight into the decision making process to mitigate the spread of disease.

Online survey marketplaces, like MTurk, can provide an effective and rapid medium for recruitment in behavioral research studies. We found that the distributions of risk associated with disease management were comparable between a sample of industry professionals and online recruits. We also identified aspects in which industry knowledge can differ throughout the presented risk dilemma. In particular, we found those with an industry background had a greater propensity to reduce uncertainty in the decision making process. Our study demonstrates the importance of validating simulated behaviors using a sample of participants with industry knowledge, in order to identify and account for potential differences that may be associated with their agricultural background. The similarities in general behavioral risk we've further investigated in this study also help validate our findings in Clark et al. (14), which tested hypotheses on a much larger sample (*N* = 1, 000) of online recruits. Our research framework highlights the viability of online marketplaces for behavioral analysis, while also demonstrating how targeted recruitment from industry stakeholders can provide additional insights into these complex decision mechanisms.

Managing the economic factors associated with disease risk management is a complex quandary. Here we quantify behavioral aspects of the decision making under risk associated with mitigating the spread of disease while maximizing profits using experimental gaming simulations. Importantly, we found that risk preferences assigned via the paired lottery choice survey were not adequate in predicting behaviors in our simulated environment. These studied behaviors and their effect on the well-being of the system as a whole should be further investigated for the promotion of healthier agricultural production networks.

## Data Availability Statement

The raw data supporting the conclusions of this article will be made available by the authors, without undue reservation.

## Ethics Statement

The studies involving human participants were reviewed and approved by University of Vermont IRB # CHRBSS-16-232-IRB. Written informed consent for participation was not required for this study in accordance with the national legislation and the institutional requirements.

## Author Contributions

EC contributed to conceptualization, data curation, formal analysis, software, visualization, and writing—original draft. SC contributed to conceptualization, investigation, methodology, project administration, supervision, and writing—review and editing. LT contributed to software and writing—review and editing. GB, NC, OL-C, and TS contributed to writing—review and editing. CK contributed to funding acquisition, project administration, and writing—review and editing. AZ contributed to conceptualization and writing—review and editing. JS contributed to investigation, project administration and writing—review and editing. All authors contributed to the article and approved the submitted version.

## Conflict of Interest

The authors declare that the research was conducted in the absence of any commercial or financial relationships that could be construed as a potential conflict of interest.
