# Supplementary material for: Emulating Agricultural Disease Management: Comparing Risk Preferences Between Industry Professionals and Online Participants Using Experimental Gaming Simulations and Paired Lottery Choice Surveys
Source: Front Vet Sci. 2021 Jan 18;7:556668. doi: 10.3389/fvets.2020.556668 (PMC7848213; doi:10.3389/fvets.2020.556668)
Supplement: Supplementary file 1 [file Data_Sheet_1.pdf]

Supplementary Material

1 DEMOGRAPHIC COMPARISON

We tested for difference in distributions of age, gender, and education between MTurk and Pork Expo participants. With regards to gender, 28 Pork expo participants identified as ‘Female’ and 22 as ‘Male’, in comparison to 31 ‘Male’ and 17 ‘Female’ from Mechanical Turk. One recruit from MTurk identified as non-binary, which was omitted from this test, as this bin would not have sufficient samples for comparison. Under these conditions, the Chi-square test did not find a measurable difference in gender distributions: $\chi^2 = 0.437, p = 0.508$. Age was binned into 3 categories {18-30, 31-40, 40+}. From the Pork Expo, 28 participants were between 18-29, 8 were between 30-39, and 14 were over the age of 40. In comparison to MTurk, we had 22 participants between 18-29, 15 were between 30-39 , and 13 were over age 40. Here, we did not see a statistical difference between these distributions: $\chi^2 = 2.887, p = 0.236$.

We did find a difference in education distributions between samples. We grouped education into 2 categories: secondary and below (i.e., up to completion of high school) and tertiary (i.e., bachelor’s degree achieved, including graduate and professional degrees earned). From our Pork Expo participants, we sampled 35 with a tertiary education and 15 with up to a secondary education. From MTurk we had 27 and 23 with a tertiary and secondary education, respectively. Here, the Chi square test reported a difference in expected frequencies between the MTurk and Expo groups: $\chi^2 = 4.967, p = 0.0258$. We note this result may be affected by our limited sample size, which forced the coarse grouping of educational responses across our samples.

Given these results, we then compared the biosecurity adoption ratings and risk lottery preferences between demographic categories. We compared differences between Expo and MTurk groups using successive KS and Mann-Whitney U tests. We did not find a significant difference between distributions for either test between demographic categories of age, education and gender.

Since the primary results did not differ between the Pork Expo and MTurk groups, we combined them to increase the sample size for demographic subgroups. When both groups were combined, we found no correlations between any demographics and game-play risk preferences. Additionally, using lottery risk preference data, we found no correlations between age and gender. However, we found a correlation between lottery risk preference and educational backgrounds (51 tertiary v. 42 secondary), where the tertiary group ($\mu = 1.31, \sigma = 0.702$) were slightly more risk preferring than the secondary ($\mu = 1.448, \sigma = 0.675$) : $U = 927.5, p = 0.0201$. This was marginally and given the number of tests performed, there is a possibility this result could be a statistical false positive. More observations may be needed to bolster the confidence in this finding. The results from each statistical analysis are summarized in Table **??**.

**Experimental Game Risk**	U statistic			*p* value		
	PE	MT	All	PE	MT	All
Gender						
Male v. Female	316.0	309.0	1283.5	0.88	0.33	0.33
Age						
20-29 v 30-39	83.5	144.5	471.0	0.28	0.53	0.21
20-29 v. 40+	166.0	125.0	563.0	0.43	0.55	0.23
30 - 39 v. 40+	46.0	95.5	291.5	0.72	0.94	0.51
Education						
Tertiary v. Secondary	273.0	265.0	1129.0	0.83	0.38	0.53
**Paired Lottery Choice Risk**	U statistic			*p* value		
	PE	MT	All	PE	MT	All
Gender						
Male v. Female	347.0	323.0	1353.0	0.44	0.19	0.13
Age						
20-29 v 30-39	133.5	113.0	532.0	0.41	0.10	0.60
20-29 v. 40+	247.5	116.5	705.0	0.16	0.36	0.74
30 - 39 v. 40+	54.0	83.0	266.5	0.91	0.51	0.38
Education						
Tertiary v. Secondary	187.0	238.5	927.5	0.10	0.15	0.04

Table S1. Demographic Statistical Comparison. Results from each two-tailed Mann Whitney U test comparing each demographic grouping with respect to experimental game simulation risk (top) and pair choice lottery risk (bottom). Results are delineated by World Pork Expo participants (PE), Mechanical Turk online recruits (MT), and their combination (All). For the most part, we did not find any significant differences per demographic, save for a marginally significant difference in pair choice lottery risk between educational groups when all participants were combined.

2 PAIRED LOTTERY CHOICE SURVEY INTERFACE

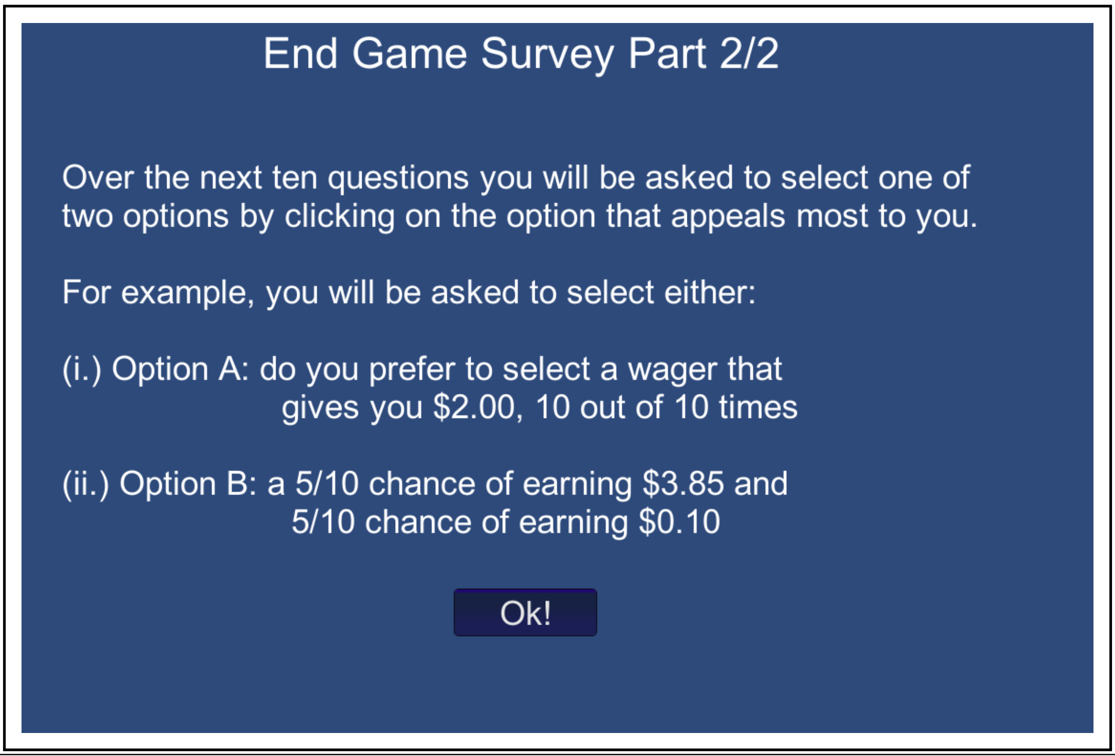

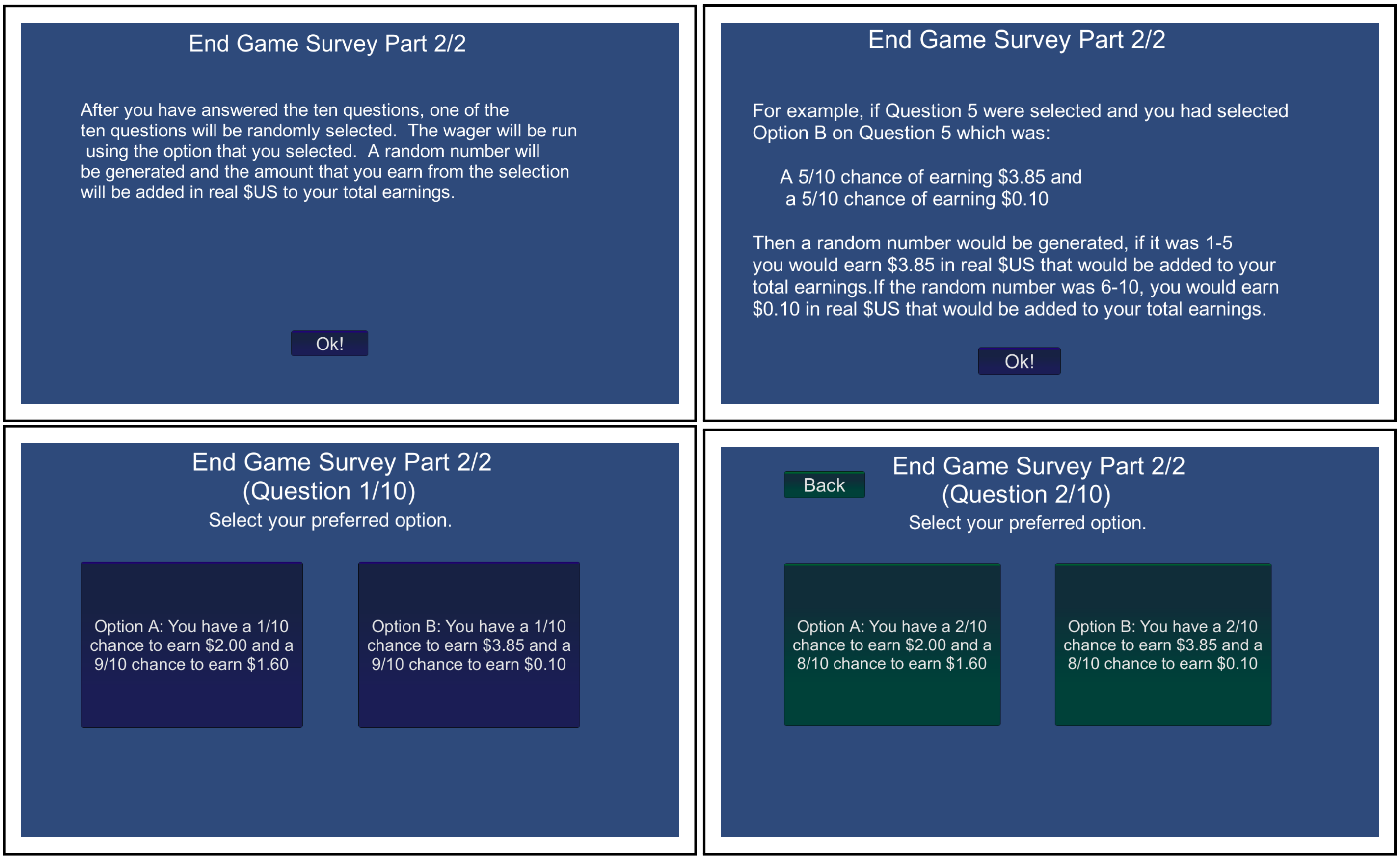
End Game Survey Part 2/2
After you have answered the ten questions, one of the ten questions will be randomly selected. The wager will be run using the option that you selected. A random number will be generated and the amount that you earn from the selection will be added in real $US to your total earnings.
Ok!
End Game Survey Part 2/2
For example, if Question 5 were selected and you had selected Option B on Question 5 which was:
A 5/10 chance of earning $3.85 and a 5/10 chance of earning $0.10
Then a random number would be generated, if it was 1-5 you would earn $3.85 in real $US that would be added to your total earnings.If the random number was 6-10, you would earn $0.10 in real $US that would be added to your total earnings.
Ok!
End Game Survey Part 2/2
(Question 1/10)
Select your preferred option.
Option A: You have a 1/10 chance to earn $2.00 and a 9/10 chance to earn $1.60
Option B: You have a 1/10 chance to earn $3.85 and a 9/10 chance to earn $0.10
Back
End Game Survey Part 2/2
(Question 2/10)
Select your preferred option.
Option A: You have a 2/10 chance to earn $2.00 and a 8/10 chance to earn $1.60
Option B: You have a 2/10 chance to earn $3.85 and a 8/10 chance to earn $0.10

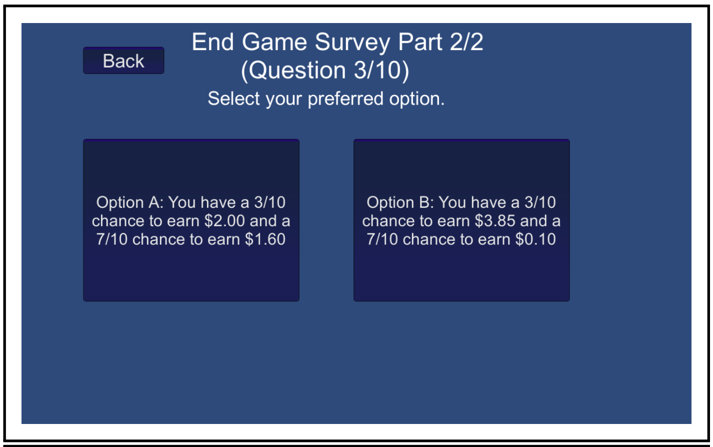
Back
End Game Survey Part 2/2
(Question 3/10)
Select your preferred option.
Option A: You have a 3/10 chance to earn $2.00 and a 7/10 chance to earn $1.60
Option B: You have a 3/10 chance to earn $3.85 and a 7/10 chance to earn $0.10

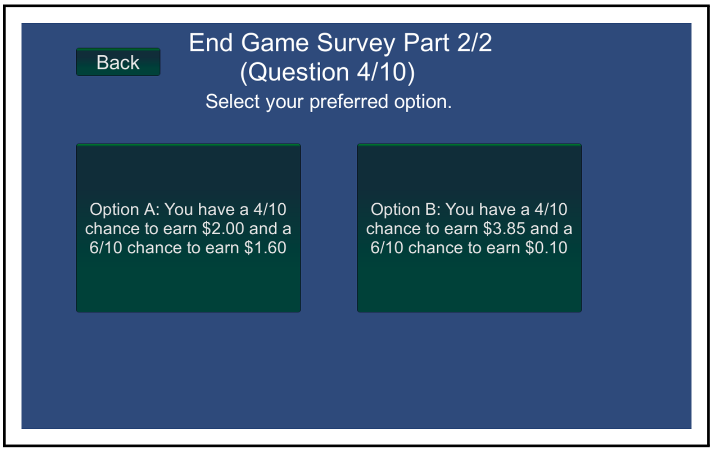
Back
End Game Survey Part 2/2
(Question 4/10)
Select your preferred option.
Option A: You have a 4/10 chance to earn $2.00 and a 6/10 chance to earn $1.60
Option B: You have a 4/10 chance to earn $3.85 and a 6/10 chance to earn $0.10

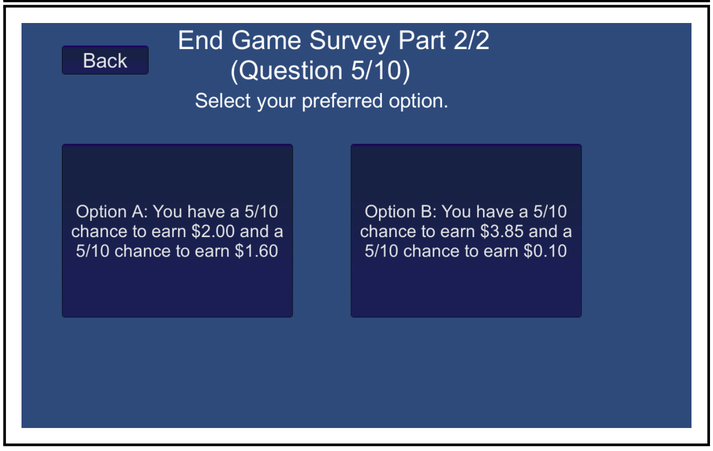
Back
End Game Survey Part 2/2
(Question 5/10)
Select your preferred option.
Option A: You have a 5/10 chance to earn $2.00 and a 5/10 chance to earn $1.60
Option B: You have a 5/10 chance to earn $3.85 and a 5/10 chance to earn $0.10

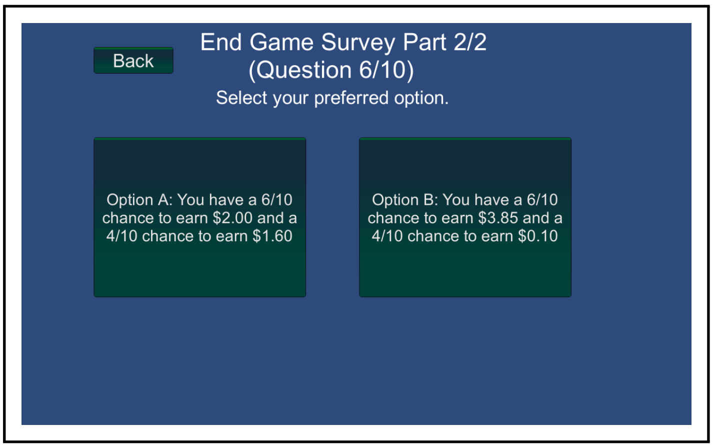
Back
End Game Survey Part 2/2
(Question 6/10)
Select your preferred option.
Option A: You have a 6/10 chance to earn $2.00 and a 4/10 chance to earn $1.60
Option B: You have a 6/10 chance to earn $3.85 and a 4/10 chance to earn $0.10

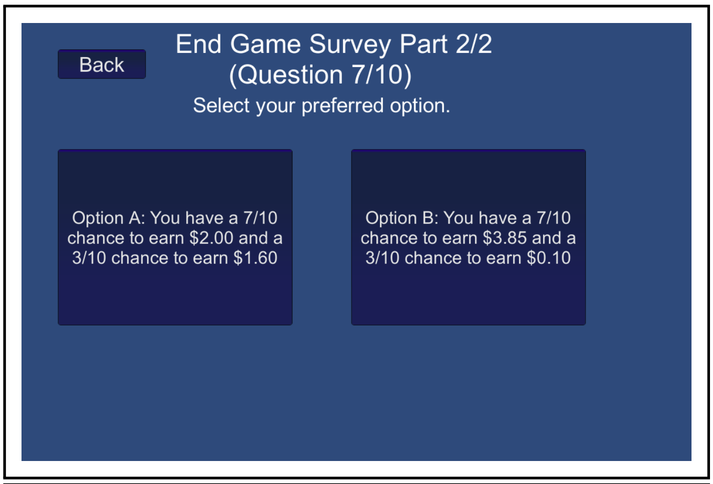
Back
End Game Survey Part 2/2
(Question 7/10)
Select your preferred option.
Option A: You have a 7/10 chance to earn $2.00 and a 3/10 chance to earn $1.60
Option B: You have a 7/10 chance to earn $3.85 and a 3/10 chance to earn $0.10

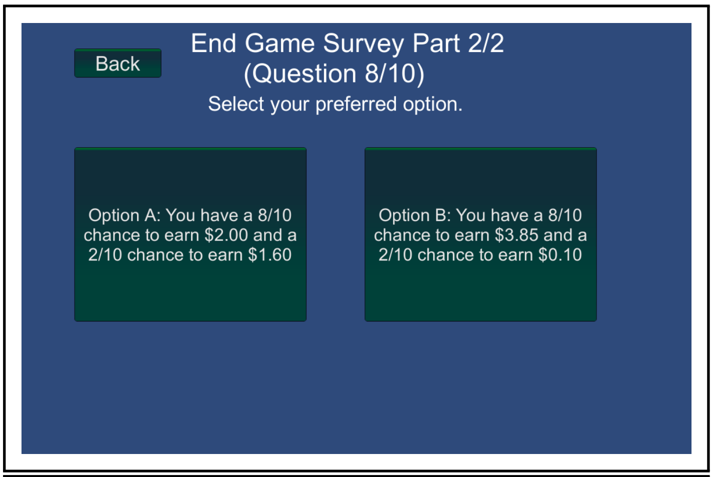
Back
End Game Survey Part 2/2
(Question 8/10)
Select your preferred option.
Option A: You have a 8/10 chance to earn $2.00 and a 2/10 chance to earn $1.60
Option B: You have a 8/10 chance to earn $3.85 and a 2/10 chance to earn $0.10

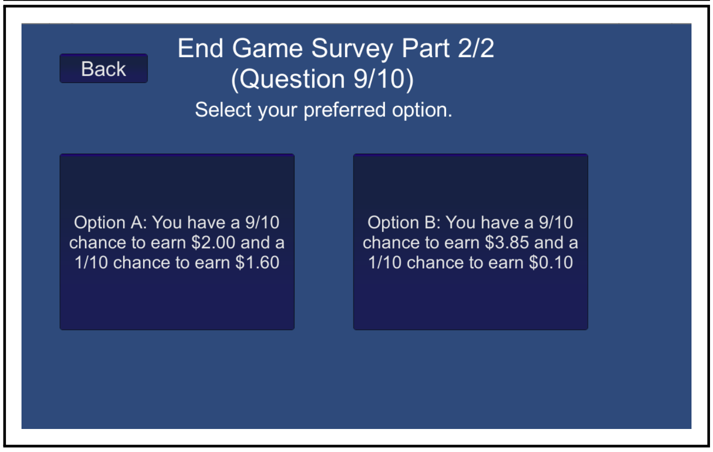
Back
End Game Survey Part 2/2
(Question 9/10)
Select your preferred option.
Option A: You have a 9/10 chance to earn $2.00 and a 1/10 chance to earn $1.60
Option B: You have a 9/10 chance to earn $3.85 and a 1/10 chance to earn $0.10

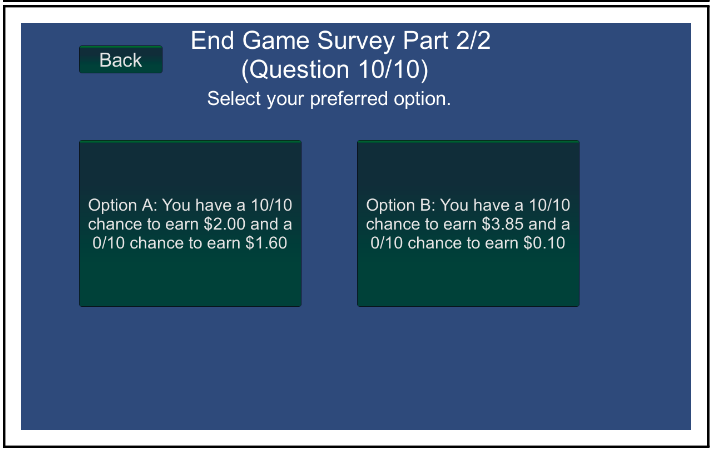
Back
End Game Survey Part 2/2
(Question 10/10)
Select your preferred option.
Option A: You have a 10/10 chance to earn $2.00 and a 0/10 chance to earn $1.60
Option B: You have a 10/10 chance to earn $3.85 and a 0/10 chance to earn $0.10
